# Relationship between Image Quality and Reproducibility of Surgical Images in 3D Digital Surgery

**DOI:** 10.3390/jcm13113051

**Published:** 2024-05-23

**Authors:** Yoshihito Sakanishi, Ayumi Usui-Ouchi, Shuu Morita, Toshiro Sakuma, Nobuyuki Ebihara

**Affiliations:** Juntendo University Urayasu Hospital, Tomioka 2-1-1, Urayasu 279-0021, Chiba, Japan; ausui@juntendo.ac.jp (A.U.-O.); s-morita@juntendo.ac.jp (S.M.); t-saku@juntendo-urayasu.jp (T.S.); ebihara@juntendo.ac.jp (N.E.)

**Keywords:** three-dimensional digital surgery, heads-up surgery, image quality, recording capacity

## Abstract

**Objectives:** Ophthalmic three-dimensional (3D) digital surgery can reproduce high-definition surgical images; however, 3D digital surgery is limited by recording capacities. We examined the relationship between the minimum image quality required to reproduce surgical images and recording capacity. **Methods:** Patients who underwent simultaneous vitrectomy and cataract surgery by the same surgeon using a 3D digital surgery system at Juntendo University Urayasu Hospital between February and October 2021 were evaluated. Various quality (Q) and frame rate (FR) settings were used for each case. Four vitreous surgeons evaluated the reproducibility of recorded images of macular manipulation for epiretinal membrane (ERM) and macular hole (MH) cases and those of peripheral retinal manipulation for rhegmatogenous retinal detachment (RRD) cases. The video bitrate and minimum settings required to reproduce surgical images and factors affecting surgical image reproducibility were examined. **Results:** A total of 129 eyes of 129 patients were observed. The minimum image quality required to reproduce surgical images was 11.67 Mbps. The Q and FR for periretinal processing and Q for macular manipulation affected surgical image reproducibility (*p* = 0.025, *p* = 0.019, and *p* = 0.07, respectively). The minimum recording settings required to obtain highly reproducible images were Q = 3 and FR = 40. The total file size for vitrectomy video recordings with these settings was as compact as 3.17 GB for 28 min. **Conclusions:** During 3D digital surgery, highly reproducible surgical images can be obtained with a small storage capacity using settings of at least Q = 3 and FR = 40.

## 1. Introduction

Traditionally, microscopes have been the standard tool used for ophthalmic surgery because they allow surgeons to observe fine details that cannot be seen with the naked eye. Recently, however, digitally assisted surgery (DAS) has gained increasing attention [1,2,3]. This approach is considered useful for cataract surgery, glaucoma surgery [4,5,6,7,8,9,10], and vitreous surgery [11,12,13,14,15,16]. DAS uses a microscope to view a three-dimensional (3D) monitor, thereby eliminating the need for an eyepiece. Additionally, DAS offers several advantages, including the ability to share the images with nonsurgical staff. During traditional microscope tube-based surgery, the assistant’s tube can be used to allow others to observe an image similar to that observed by the surgeon; otherwise, they are limited to viewing a two-dimensional (2D) image on a monitor in the operating room, which offers a different perspective from that of the surgeon. In contrast, DAS allows everyone in the operating room to view the same 3D image, facilitating a better understanding of the surgical procedure. When using conventional optical tubes, many surgeons experience musculoskeletal issues because of the need to lean slightly forward [17]. However, DAS enables surgeons to operate in a comfortable position because it eliminates the need to peer into a tube. Moreover, DAS enables surgery under low-light conditions. Digital surgery systems have the capability to enhance images with greater sensitivity than the actual light intensity, ensuring consistent brightness levels, whether in low-light or normal-light conditions during surgery [4,5]. For example, strabismus surgery can be performed without the use of the microscope light [18]. Another significant advantage of DAS is its ability to record highly detailed 3D surgical images that can be reviewed postoperatively. This feature enables the reproduction and review of the same images observed during surgery; this is not possible with conventional 2D surgical video recording, and this represents a significant advantage for surgical education. However, high-definition surgical videos require a large amount of storage space. The NGENUITY 3D digital surgery system (Alcon, Fort Worth, TX, USA) used during DAS requires 12–15 GB of storage space to record 28 min of surgical video using the default video recording settings, which is several times greater than that required for conventional 2D surgical video recording. The need for such a large recording capacity has resulted in numerous challenges, including elevated costs and limited accessibility to recording media. Several parameters can be adjusted to obtain the desired quality of the recorded video; however, the capacity required for the recorded video varies depending on these parameters. Optimizing these settings could solve the aforementioned cost and accessibility issues without requiring unnecessarily large storage capacities. Efficient storage solutions could broaden the accessibility of DAS, thus making its benefits available in a wider range of healthcare settings, including those with limited storage capacities. Subsequently, this could enhance surgical outcomes and educational opportunities. However, the balance between maintaining high-quality surgical images and efficiently managing the storage capacity is not well understood. As mentioned above, recording in high-quality, high-definition settings can closely replicate surgical images observed during the procedure, albeit at the expense of significant storage capacity. Conversely, opting for lower-quality recording settings reduces storage requirements but compromises the reproducibility of surgical images, thus underutilizing the advantages of 3D surgery. It is crucial to ascertain the optimal balance of recording settings that maintain surgical reproducibility while managing storage demands effectively. Therefore, we investigated the relationship between minimum video settings required to reproduce surgical images and storage capacity.

## 2. Materials and Methods

### 2.1. Subjects

This study included patients who underwent simultaneous vitrectomy and cataract surgery performed by the same skilled surgeon (Y.S.) using NGENUITY version 1.3 (Alcon) at Juntendo University Urayasu Hospital between 1 February and 31 October 2021. The eligibility criteria included age > 18 years and good intraoperative fundus visibility. Patients who underwent concomitant buckling or glaucoma surgery and those with corneal opacities were excluded from the study.

This retrospective study was reviewed and approved by the Juntendo University Ethics Committee (E21-0180, approval Date: 1 August 2021). The patients’ identifying characteristics were anonymized before the video was extracted. Consent was obtained using an opt-out system. The study was conducted according to the guidelines of the Declaration of Helsinki.

### 2.2. Video Recording Settings

The video recording settings of NGENUITY (Alcon) allow for adjustments to two parameters: the image quality (Q) and frame rate (FR). The FR indicates the number of frames per second (fps). The Q parameter ranges from 0 to 10, and the FR can be set between 30 and 58 fps. Q was adjusted in six increments from 0 to 10 using two steps, and FR was adjusted in six increments from 30 to 55 using five steps. The video bitrate (expressed in Mbps), which represents the amount of data processed per second, was used as a metric for video quality and calculated as follows: video bitrate = video capacity (MB)/video duration (s)/0.25.

### 2.3. Surgical Environment

All surgeries were performed using the NGENUITY 3D digital surgery system (version 1.3; Alcon) in combination with a Lumera 700 microscope (Zeiss, Oberkochen, Germany), a wide-angle fundus viewing system, and a 27 G Constellation vitrectomy machine (Alcon) operated at 20,000 rpm. The NGENUITY (Alcon) aperture was set to 30%.

### 2.4. Items for Consideration

Four vitreous surgeons viewed the 3D images recorded by NGENUITY (Alcon) using various video recording settings, as previously described, encompassing cases involving macular manipulation for epiretinal membrane (ERM) and macular hole (MH), as well as peripheral retinal manipulation for rhegmatogenous retinal detachment (RRD). During this procedure, macular manipulation was performed using chandelier lighting supplemented by a handheld light pipe for targeted macular illumination during vitreous forceps manipulation. For peripheral retinal manipulation, chandelier lighting illuminated the fundus coupled with a wide-angle viewing system. Scleral depression was applied with one hand while the other operated a vitreous cutter for peripheral vitreous removal. Then, the surgeons blindly evaluated the surgical reproducibility using the following 5-point scale:

1. The surgical content was almost unrecognizable.
2. I can barely understand what type of surgery is being performed.
3. I can understand what is being performed during surgery, but I cannot perform the surgery based on the video.
4. I can understand the details of the surgery sufficiently well to perform it because of the good image quality.
5. The image quality was comparable to that of normal surgery and allowed standard surgical performance.

A score of 4 was considered acceptable. The average standard value for “surgical reproduction” was considered 3.75, indicating that three of four evaluators rated the image quality as 4, and one of those four evaluators rated it as 3.

The primary outcomes of this study were the correlation between the video bitrate and reproducibility ratings of macular manipulation for ERM/MH cases and periretinal processing for RRD cases. Additionally, the study aimed to explore the relationship between video bitrate and Q and FR settings across all patients and to identify Q and FR settings that could potentially surpass the threshold video bitrate necessary for accurate reproduction. To investigate this relationship, we examined whether there was a correlation between the video bitrate and surgical reproducibility of images of macular manipulation for ERM and MH cases and images of peripheral retinal processing for RRD cases.

The secondary outcome was the relationship between surgical reproducibility and video bitrates. Factors that affected the surgical reproducibility of ERM/MH and RRD were also examined.

### 2.5. Statistical Analyses

Data were analyzed using the Statistical Package for the Social Sciences (version 26; SPSS Inc., Chicago, IL, USA). To evaluate the relationship between surgical reproducibility and the video bitrate (the primary outcome), a cutoff value was established using the receiver operating characteristic curve and Youden’s index. The correlation between the video bitrate and reproducibility ratings (a secondary outcome) was assessed using Spearman’s rank correlation coefficient. Factors that affected surgical reproducibility were analyzed using binomial logistic regression.

## 3. Results

We evaluated 129 eyes of 129 patients. The details of all cases are presented in Table 1.

### 3.1. Correlation between Video Bitrate and Reproducibility Ratings for Macular Manipulation of ERM/MH Cases and Periretinal Processing of RRD Cases

**Figure 1:**
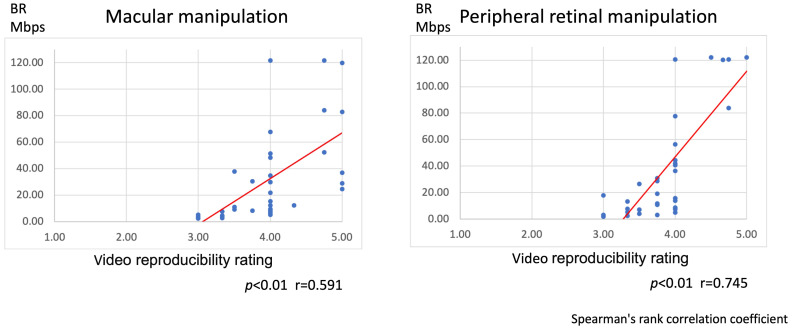
Correlation between the video bitrate and video reproducibility ratings. A positive correlation was observed between the video bitrate and effective video reproducibility for both macular manipulation and peripheral retinal manipulation. The red line represents approximate straight line.

We observed a positive correlation between the image bitrate and image reproducibility ratings for both macular manipulation and peripheral retinal processing (Figure 1).

### 3.2. Correlation between Video Bitrate and Reproducibility Ratings

**Figure 2:**
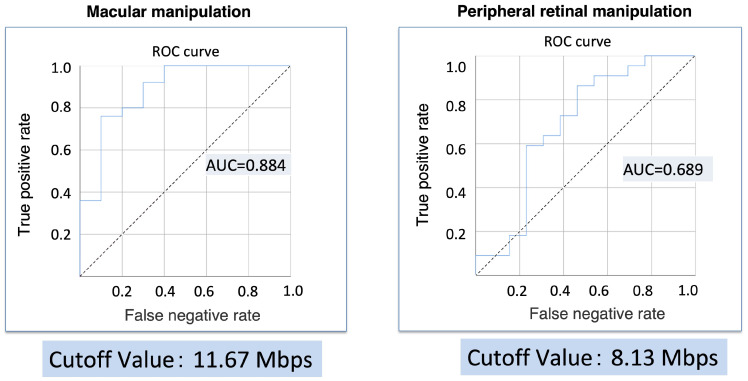
Relationship between surgical reproducibility and the video bitrate.

Additionally, the evaluation of the relationship between surgical reproducibility and the video bitrate (the primary outcome) indicated cutoff values of 11.67 Mbps for macular manipulation and 8.13 Mbps for peripheral retinal manipulation (Figure 2). These video bitrates were observed when the FR and Q settings were adjusted independently.

The minimum video bit rate required for surgical video reproduction was 11.67 Mbps for macular manipulation and 8.13 Mbps for peripheral processing. The video bitrate required for macular manipulation was higher than that required for peripheral retinal manipulation.

### 3.3. Relationship between the FR and Video Bitrate

**Figure 3:**
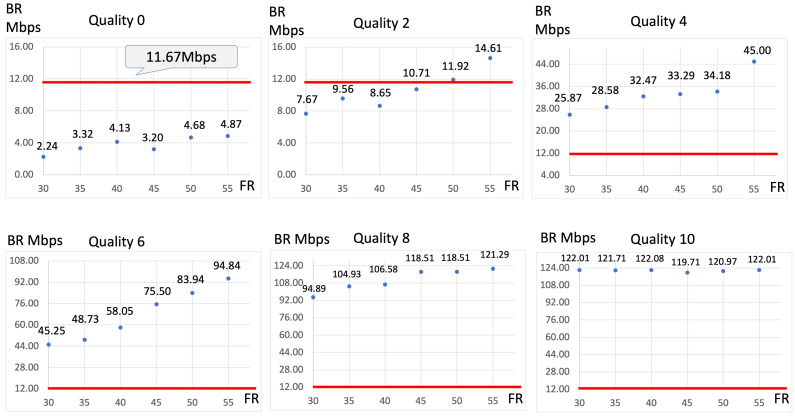
Relationship between the frame rate (FR) and video bitrate at each video quality (Q) setting. The video bitrate exceeds 11.67 Mbps when the FR is set to ≥50 fps at Q = 2. At Q = 4, the video bitrate exceeds 11.67 Mbps when the FR is set to 30 fps. The red line indicates the position where the video bitrate is 11.67 Mbps.

**Figure 4:**
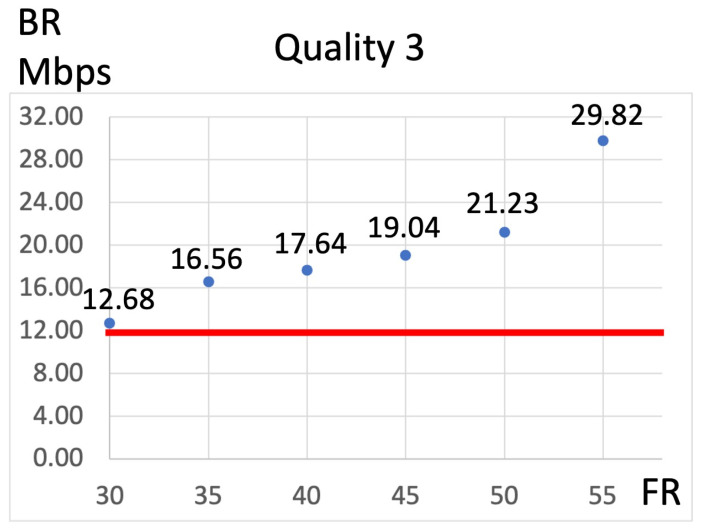
Relationship between the frame rate (FR) and video bitrate with quality (Q) set at 3. The video bitrate at Q = 3 exceeds 11.67 Mbps when the FR is set to 30 fps. The red line indicates the position where the video bitrate is 11.67 Mbps.

Figure 3 illustrates the relationship between the FR and video bitrate under a consistent Q setting. Two video bitrates were identified as necessary for adequate surgical visualization, with the highest being 11.67 Mbps. This threshold consistently surpassed all FRs when Q was set to 0. However, when Q was set to 2, none of the FRs reached this threshold. In contrast, with Q values equal to or greater than 4, the threshold of 11.67 Mbps was exceeded at every FR. Specifically, with Q set to 2, the threshold was surpassed at all FRs greater than 50 fps, while with Q values of 4 or higher, the threshold was exceeded at all FRs. Consequently, when recalculating the video bitrate with the FR set in conjunction with Q = 3, the 11.67 Mbps threshold was exceeded at an FR of 30 fps (Figure 4).

### 3.4. Relationship between Q and the Video Bitrate

**Figure 5:**
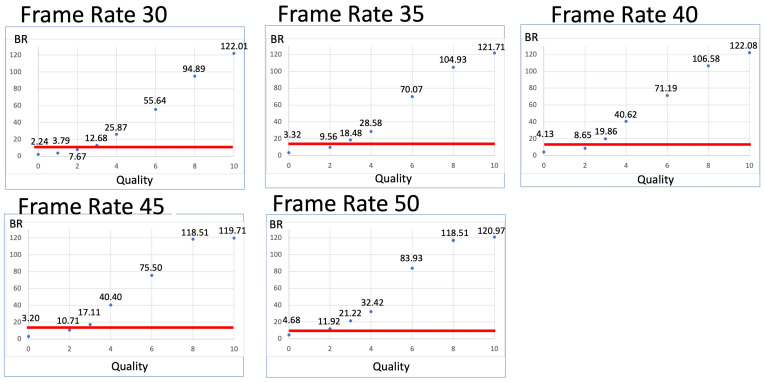
Relationship between video quality (Q) and the bitrate at each frame rate (FR). Video bitrates exceeding 11.67 Mbps are observed at Q ≥ 3 for FRs of 30, 35, 40, and 45 fps and at Q ≥ 2 for an FR of 50 fps. The red line indicates the position where the video bitrate is 11.67 Mbps.

Figure 5 presents the relationship between Q and the video bitrate when a constant FR is maintained. The video bitrate of 11.67 Mbps was exceeded when Q ≥ 3 for FRs of 30, 35, 40, and 45 fps. Additionally, Q ≥ 2 was sufficient to exceed the 11.67 Mbps threshold at an FR of 50 fps.

The Q significantly correlated with factors that affected the surgical reproducibility of macular manipulation of ERM and MH cases. Furthermore, both Q and FR were independently and significantly associated with factors that affected the surgical reproducibility of the peripapillary retinal manipulation of RRD cases (Table 2).

## 4. Discussion

In this study, we examined the reproduction of surgical images recorded using a 3D monitor and their corresponding video capacities. We found that the minimum video bitrates required to reproduce these surgical images were 11.67 Mbps for macular manipulation and 8.13 Mbps for peripheral manipulation. Although not statistically examined, macular manipulation required a higher video bitrate than periretinal processing, suggesting that macular manipulation requires a higher resolution for optimal imaging. Several studies have highlighted the usefulness of DAS in vitreous surgery, many of which have suggested DAS to be more advantageous than conventional specular microscopy, especially for macular manipulation [19,20]. This preference may be attributed to the image processing capabilities of DAS [14,21], which are particularly beneficial for macular manipulation.

To the best of our knowledge, there are no reports on the reproducibility of recorded surgical images, which is a notable feature of DAS. In traditional surgery utilizing a microscope, the recorded images are typically in 2D format. Consequently, during post-surgical review, these images fail to accurately replicate the original surgical perspective as they are presented solely as flat 2D images. Before the advent of DAS, the concept of surgical reproducibility did not exist; therefore, it was not a subject of debate. However, DAS allows surgeons to postoperatively view images in 3D. These images closely resemble the experience of live surgery. This study may, therefore, serve as a foundation for future discussions regarding surgical reproducibility.

This study is significant because it determined the minimum image quality required to record 3D surgical images. Since 3D surgical images are typically high-definition, they require substantial storage space. However, the use of the minimum video bitrate required to reproduce quality surgical images reduces the need for extensive recording space. Thus, adopting the settings identified in this study to maximize the benefits of recorded images would improve the accessibility of DAS. Furthermore, with the emergence of various new surgical approaches utilizing DAS, recording these procedures can facilitate their wider dissemination. In this context, this study is expected to play a pivotal role in advancing the utilization and dissemination of DAS technology [22,23,24].

Q was a significant factor for both macular and peripheral procedures, and the FR was particularly pertinent to peripheral procedures. Although various vitrectomy techniques exist, our study focused on these two crucial techniques. During macular manipulation, intricate observations are required for localizing the ERM and discerning the stained inner limiting membrane; this explains why a higher Q is correlated with better surgical reproduction. In contrast, the FR was less impactful on macular manipulation, possibly because such procedures do not involve rapid movements or vitreous flapping, thus allowing for high reproducibility even at lower FRs.

During peripheral manipulation, which evaluates the behavior of the fluttering peripheral retina in retinal detachment, we found that both Q and FR are crucial. Additionally, the role of FR in peripheral manipulation is significant because the fine movements of the fluttering retina necessitate a higher FR for accurate representation.

Our findings highlight the minimum video bitrate and appropriate video recording settings required to reproduce 3D surgical videos. When macular and peripheral processing were combined, the minimum bitrate was 11.67 Mbps. When both the Q and FR were set above the minimum levels, specifically Q = 3 and FR = 40 fps, the minimum bitrate was exceeded. This setting reduced the recording capacity to 3.17 GB for 28 min, which is lower than the typical 4.2 GB required for 28 min of a standard 2D recording. Traditionally, 3D videos were recorded as two separate 2D videos, effectively doubling the storage capacity required. However, the minimum 3D image quality identified in this study was found to be less than half that of standard 2D images.

Several factors can ensure the reproducibility of macular manipulation and other procedures. One of these factors is image optimization through high dynamic resolution. The software used in this study optimized various image qualities, such as the contrast between bright and dark areas, using the high dynamic resolution function. Additionally, because surgeons perform procedures while directly viewing the monitor, they can record optimally focused images by adjusting their focus. These factors allow NGENUITY (Alcon) to record highly reproducible images. While standard 2D videos may not be considered of minimal quality and direct comparison may not be entirely equitable, it is evident that the reduced capacity required for 3D surgical videos is still significant enough to justify their utilization.

This study had some limitations. First, individual differences in 3D high-definition ultra-widefield surgical monitors may exist. This study used only one type of monitor; however, individual variations may affect the results of future studies. Therefore, similar studies should include different monitors. Second, brightness, other lighting settings, and the type of lighting varied among surgeons. In this study, both chandelier and handheld lighting were employed with set brightness settings; however, it is necessary to conduct tests under a range of conditions. Many facilities use both lighting types. Since we applied standard lighting conditions in our study, we believe that these conditions are generally applicable to other facilities. Furthermore, as NGENUITY (Alcon) continues to evolve and acquire new features, such as contrast enhancement, it will be important to consider the impact on storage capacity when leveraging these functions.

## 5. Conclusions

Our study determined the minimum recording quality settings necessary for effective video reproduction on 3D high-definition ultra-widefield surgical monitors. Identifying the minimum video bitrate required to reproduce a sufficient surgical image is a practical approach to effectively reduce the required storage space.

## Figures and Tables

**Table 1:** Cases included in this study.

Items	N = 129
Sex (men/women)	76/53
Age (years) ± SD	67.3 ± 12.3
Disease	Eyes
Epiretinal membrane	22
Macular hole	13
Rhegmatogenous retinal detachment	33
Intraocular lens dislocation	12
Proliferative diabetic retinopathy	24
Others	25

SD; standard deviation.

**Table 2:** Factors related to surgical reproducibility.

Macular Manipulation			
	*p* Value	Odds Ratio	95% CI
Quality	0.007 *	3.618	1.423–9.198
Frame rate	0.942	0.995	0.880–1.126
**Peripheral Processing**			
	***p* Value**	**Odds Ratio**	**95% CI**
Quality	0.025 *	3.612	1.179–11.063
Frame rate	0.019 *	1.271	1.040–1.554

Binomial logistic analysis * *p* < 0.05; CI; confidence interval.

## Data Availability

This material is available upon request to interested researchers.
